# Enhancement of Thermoelectric Performance for CuCl Doped P-Type Cu_2_Sn_0.7_Co_0.3_S_3_

**DOI:** 10.3390/ma16062395

**Published:** 2023-03-16

**Authors:** Dong-Liang Shi, Kwok-Ho Lam

**Affiliations:** 1 Department of Electrical Engineering, Research Institute of Smart Energy, The Hong Kong Polytechnic University, Hong Kong; 2 Centre for Medical and Industrial Ultrasonics, James Watt School of Engineering, University of Glasgow, Glasgow G12 8QQ, Scotland, UK

**Keywords:** thermoelectric, Cu_2_SnS_3_, repeatability, energy conversion

## Abstract

Cu_2_SnS_3_ (CSS) has gained great attraction due to its constitutive earth-abundant elements and intrinsic low lattice thermal conductivity, $\kappa_{l}$, potentially providing high quality factor, B, and high zT value. However, the lack of band convergence is the bottleneck to enhancing the thermoelectric performance of Cu_2_SnS_3_ when performing the band engineering. To study the doping effect on the band structure and the thermoelectric performance, the composite Cu_2_Sn_0.7_Co_0.3_S_3_-*x*CuCl (*x* = 0, 0.1, 0.2, 0.3) (CSCS-*x*CuCl) has been investigated for the first time. The samples showed excellent data repeatability at high temperatures of up to 700 K. It was found that CuCl could compensate the Cu loss, enhance the phonon scattering and minimize the adverse effect on the power factor, PF. The ultralow lattice thermal conductivity could reach 0.38 W m^−1^ K^−1^ for the nominal composition of CSCS-0.3CuCl at 700 K. A peak zT of 0.56 (evaluated with no cold finger effect) was realized at 700 K when *x* = 0.3, which is almost double the performance of pristine samples.

## 1. Introduction

Cu_2_SnS_3_ (CSS) possesses a complex crystal structure with low-temperature tetragonal, monoclinic, and triclinic phases [1,2,3,4,5,6,7,8], and the cubic phase has a high transition temperature (>800 K). With the monoclinic phase structure, the theoretical bandgap of CSS was estimated to be 0.88 eV by monitoring the exchange energy of the Heydt-Scuseria-Ernzerhof (HSE) function and varying the Hartree-Fock (HF) part [9,10]. More specifically, the valance band maximum (VBM) is composed of Cu 3d and S 3p components, while the conduction band minimum (CBM) is composed of 5s and 3p components [9]. It should be noted that the carrier concentration of the pristine CSS is low at a level of ~10^20^ cm^−3^, which is far less than the optimized level (~10^21^ cm^−3^) [11]. As the Fermi level of the pristine CSS locates near the edge of VBM, the doping at Sn sites could shift the Fermi level downwards to enhance the carrier concentration [11]. Thus, the enhancement of thermoelectric efficiency can be realized. Up to now, most of the reported thermoelectric studies on CSS-based materials were based on a low symmetry phase.

Nonetheless, due to flat bands at the VBM site, the effective mass, $m^{*}$, increases with the simultaneous decrease in carrier mobility, μ, inhibiting the enhancement of the thermoelectric performance. Moreover, the lack of band convergence (alignment of energy bands [12]) for the pristine CSS also hinders the further improvement of this promising thermoelectric material. So far, doping at Sn sites with the foreign atom (Zn [11], In [13], Co [14], Cu [15]) has been studied systematically. For instance, the substitution to Sn sites brought a large enhancement of hole carrier concentration; however, it heavily dampened the mobility. Furthermore, except for the alloying study at both the Cu and Sn sites, composite inclusions such as SnS [16] were explored by Riya et al. in the low-temperature range. The nanostructure of the CSS phase has also been successfully synthesized by the wet ball milling method [17,18].

The CSS structures (cubic, tetragonal, and monoclinic) are all based on a corner-sharing tetrahedron with Cu and Sn positioned at the tetrahedral site coordinated with 4S atoms at the corners, and thus are recognized as diamond-like ternary compounds. However, the arrangement of metal atoms inside the S sublattice is different from each other. In the monoclinic structure, Cu and Sn atoms fully occupy the separated 2a tetrahedral sites in an orderly manner. While in the tetragonal structure, there are three different occupations for the tetrahedral sites, i.e., the 2a tetrahedral sites (accounting for ¼ of total) are occupied by Cu atoms only, and the 4d and 2b sites are occupied by the composite atoms M1 (43.6 at% Sn + 56.4 at% Cu) and M2 (46.3 at% Sn + 53.7 at% Cu), respectively. In the cubic structure, the tetrahedral sites become equivalent, and fully occupied by composite atoms M3 (66.7 at% Cu + 33.3 at% Sn), which means a high-degree disordering of Cu and Sn atoms at these sites [11]. The phase formation depends on the arrangement of these cations and temperature. The monoclinic phase is a low-temperature stable structure, while the cubic phase is a high-temperature isomorphic phase with a cubic ZnS structure [19]. The tetragonal CSS is an intermediate phase between the high-temperature cubic and low-temperature monoclinic phases. As there is a very slight difference between cubic and monoclinic CSS lattice parameters, the alloying with Cu or Sn site would easily change in between these phase structures.

Up to now, for most reported modified CSS or CSS composites, the thermoelectric efficiency could reach decent values of 0.3–0.5 at a temperature of ~700 K. The study of grain size on thermoelectric performance was performed by Lohani [20]. The nanosized grain (~12 nm) shows the highest *zT* value of 0.4 at 650 K. By manipulating the twin boundary, an enhanced thermoelectric performance of CSS was obtained by Zhou’s group [21], with a *zT* value of 0.36 at 700 K. The effect of porosity on thermoelectric performance was studied systematically by Lohani, with a *zT* value of 0.34 at 700 K [22]. The further enhancement seems to cease due to the following reasons: (1) From the band structure analysis, the divergence of bands at Γ point proves a semiconductor with low degeneracy, thus the *PF* is low for the pure CSS phase [23]; (2) The complicated phase composition issue: the strong dependence of the phase structure of CSS on the composition brings the difficulty of obtaining a pure phase structure, leading to the difficulty to target on one single phase to study the enhancement of *zT*. A record *zT* of ~0.85 has been reported by Zhao et al., through doping with Co using the melting method [14]. However, the induced high S vapor pressure during the melting progress would make it difficult to reach the phase equilibrium during the synthesis process. Furthermore, the high vapor pressure of S would surpass the limit of the quartz tube, leading to a safety issue that would hinder the mass production of cost-effective thermoelectrics. In this study, the traditional ball milling and hot press sintering method are employed to enhance the thermoelectric performance of Co-modified CSS via doping CuCl. The dopant of CuCl plays an important role in compensating copper sources and performing chlorine doping [24]. Finally, the phase structure, thermoelectric performance, and acoustic properties have been explored systematically.

## 2. Experimental Parts

### 2.1. Synthesis Method

High purity CuCl (99.9%), S (99.99%), Sn (99.9%, 300 mesh), Cu (99.9%, 100 nm) and Co (99.99%) powders were weighed according to the designated nominal composition of CSCS-*x*CuCl (*x* = 0, 0.1, 0.2, 0.3). The raw materials were directly transferred into a stainless-steel jar for a 30-min alloying process during the impact grinder. The synthesized powder was then weighed and transferred into a graphite mold to perform a hot press sintering process. The pressure was set to 80 MPa at 873 K for 1 h under a vacuum condition of below 0.01 Pa. After sintering, the pressure was released immediately, and the sample was cooled in a vacuum with the furnace. The sintered disc was obtained with a diameter of about 12.5 mm, and the thickness was polished to 1 mm.

### 2.2. Characterizations

X-ray diffraction (XRD) with Cu K_α_ radiation (D8 Advance, Bruker, Karlsruhe, Germany, λ = 1.5418 Å) was used to characterize the phase composition of samples. Scanning electron microscope (SEM) and energy dispersive X-ray (EDX) characterization were conducted on the polished surface of the sintered sample by using the VEGA3 (TESCAN, Brno, Czech Republic). Netzsch SBA458 (Netsch, Selb, Germany) was used to measure the electrical transport properties (i.e., electrical conductivity, σ and Seebeck coefficient, *S*). Total thermal conductivity, *κ* was calculated according to the equation *κ* = *D*⋅*C*p⋅*ρ*, where thermal diffusivity, *D*, was measured with a laser flash apparatus (LFA457, Netsch, Selb, Germany). The measurement of specific heat capacity, *C*p, was conducted on fragments of the sintered sample using the sapphire method with the Mettler Toledo TGA/DSC3+ (Mettler Toledo, Columbus, OH, USA) instrument. Density, *ρ*, was measured using the Archimedes method. The sound speeds were determined by the homemade instrument using the Olympus 5-MHz longitudinal transducer and 5-MHz transverse transducer along with the pulse-echo method.

## 3. Results and Discussion

**Figure 1:**
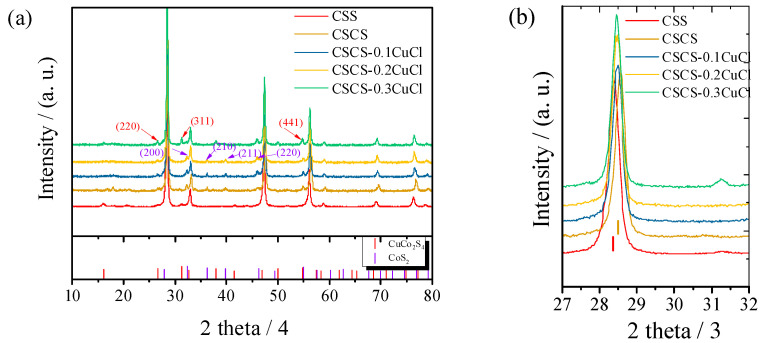
(**a**) Powder X-ray diffraction (XRD) patterns for CSCS-*x*CuCl (*x* = 0, 0.1, 0.2, 0.3) samples and the pristine phase CSS. (**b**) enlarged XRD profile in the range from 27° to 32°.

**Figure 2:**
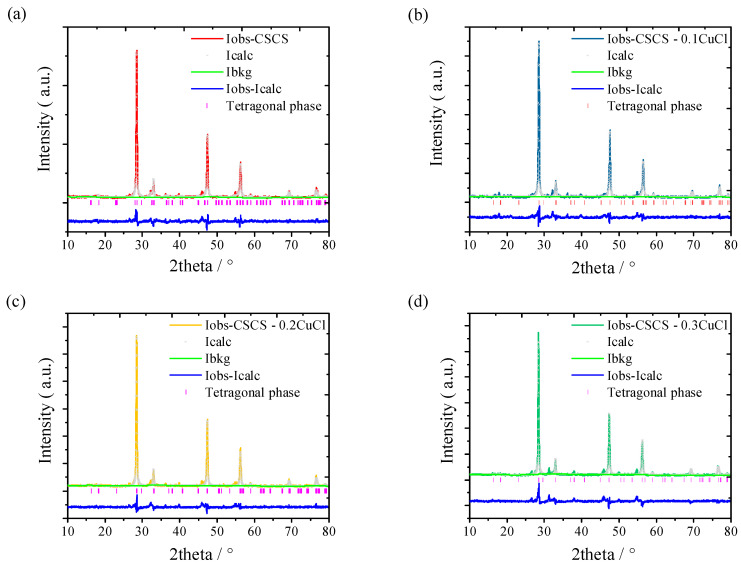

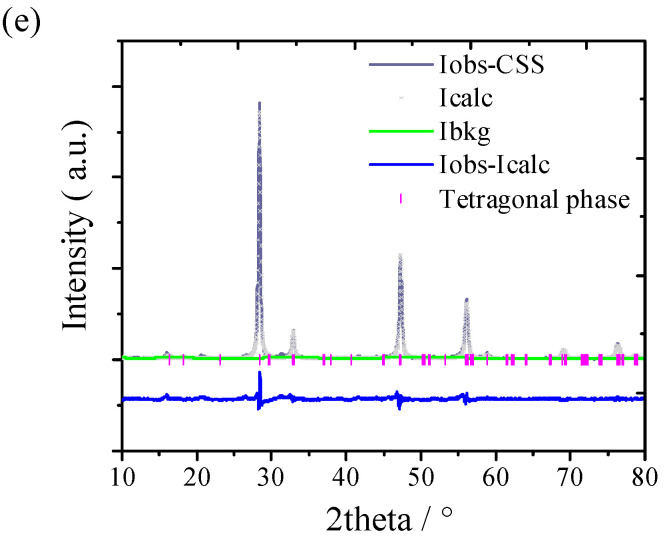
Refinement results for (**a**) CSCS, (**b**) CSCS—0.1CuCl, (**c**) CSCS—0.2CuCl, (**d**) CSCS—0.3CuCl samples and (**e**) pristine sample CSS, respectively.

**Figure 3:**
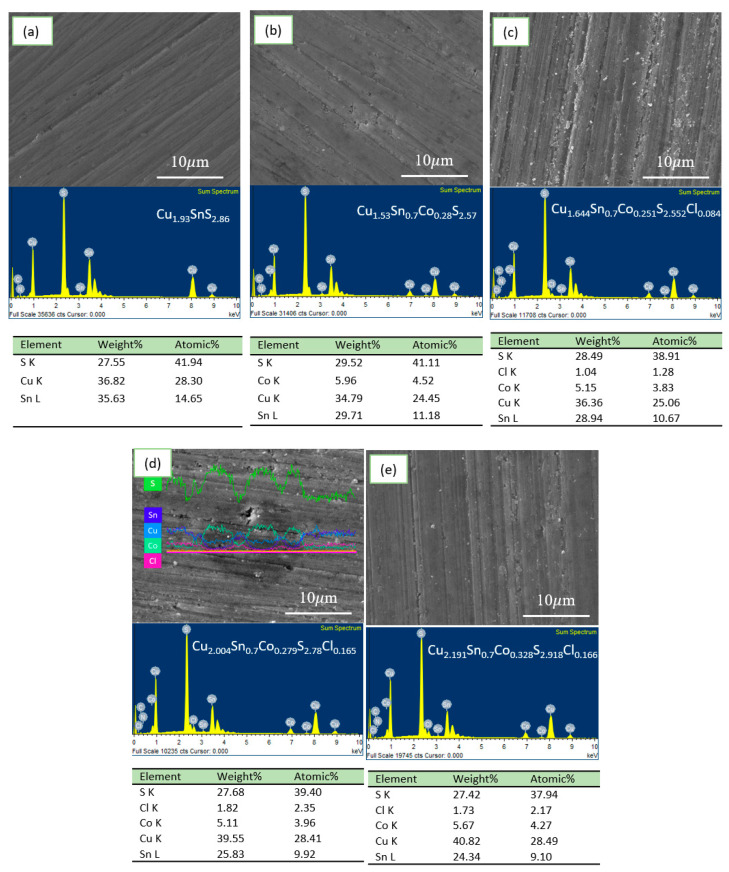
(**a**–**e**) Scanning electron microscopy (SEM) and energy-dispersive X-ray spectra (EDX) analysis for the polished surface of the sample. (**d**) The linear EDX analysis for sample CSCS-0.2CuCl.

The phase structure of all sintered samples was analyzed by performing powder XRD from 10° to 80° (Figure 1 and Figure 2). It was found that the pristine CSS sample was shown to correspond well to the tetragonal phase (PDF#04-009-7947, I$\bar{4}$2m), which is quite different compared to the reported work [11,13,15,17]. This is due to the existence of copper and sulfur vacancies, which is demonstrated by the EDX results shown in Figure 2a. The copper vacancy originates from the Cu loss during the mechanical alloying progress, since the ball milling machine utilized in this experiment only performs the impact grinder and blender at the bottom of the vial. In addition, the copper raw material is nanosized and light compared to Sn, which is likely to attach to the cap. Compared to our milling machine, others such as the Mixer/Mill 8000 M model performs as an impact grinder, and the grinding vial is shaken back and forth in a vigorous motion, which occurs on all inner surfaces and leads to a uniform blending and alloying process. Traces of copper and sulfur would stick to the cap, leading to the deviation of the composition from Cu_2_SnS_3_ (Figure 3a). With Co doping, a sudden change of peak from 28.40° to 28.56° (Figure 1b) indicates that the phase microstructure varies. In detail, the unit cell shrinks with the Co doping due to the smaller radius of Co^2+^ (67 pm) compared to Sn^4+^ (69 pm). By performing XRD refinement using GSAS software [18], the shrinkage of the lattice parameters and cell volume can be verified and shown in Table 1. In addition, a trace of the CoS_2_ (PDF#04-003-2294) with space group (Pa$\bar{3}$) could be detected with impurity peaks in the vicinity of 27.88°, 32.30°, 39.84° and 46.33° for CSCS. Moreover, cubic phase SnS (PDF#04-004-8426) can also be detected. With the introduction of CuCl, the lattice parameters for the tetragonal phase enlarges especially for the a and b axis when 10% CuCl is doped. As the amount of dopant CuCl increased from 20% to 30%, the lattice parameters increased and reached saturation with the cell volume to 318.819 Å^3^. Specifically, the cubic impurity phase CuCo_2_S_4_ (PDF#04-005-8680) is detected when 30% CuCl is doped in the phase.

Figure 3 shows the SEM image and EDX analysis on the surface of all the sintered samples. All of the atomic percentage was modified with Sn content as 1 and 0.7 for convenient comparison. For the CSS sample, the quantitative analysis of the composition is Cu_1.93_SnS_2.86_, which deviates from the nominal formula with decent copper and sulfur vacancies. The origin of copper vacancy and sulfur vacancy was discussed previously. With the introduction of Co, the copper and sulfur vacancies increase, which is particularly due to the formation of the impurity phase, CoS_2,_ and Cu_1.8_S. The increased copper vacancies would largely increase the carrier concentration and influence electron transport. To compensate for the loss of Cu, the CuCl dopant plays a critical role in decreasing the Cu vacancies. For the nominal composition Cu_2_Sn_0.7_Co_0.3_S_3_-0.1CuCl, the EDX analysis results show a composition Cu_1.644_Sn_0.7_Co_0.28_S_2.552_Cl_0.084_. The source of Cu mainly comes from the dopant CuCl. In addition, with the increasing content of CuCl, the copper vacancy decreases. When *x* equals 0.2, the composition is analyzed to be Cu_2.004_Sn_0.7_Co_0.279_S_2.78_Cl_0.165_, which shows excess Cu and a similar sulfur content compared to the CSCS sample. In Figure 3d, the linear scan of EDX analysis for the sample CSCS-0.2CuCl shows the change of the relative content for each element regarding the morphology. The black ball shape with the size of approximately 10 μm displays a high content of sulfur, cobalt, and relatively low copper compared to the matrix. The impurity is likely to be the CoS_2_ phase, which is consistent with the XRD analysis.

**Figure 4:**
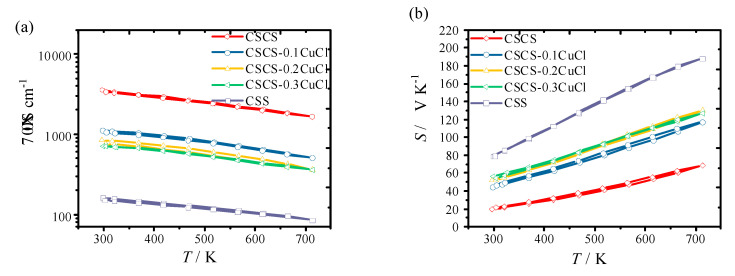

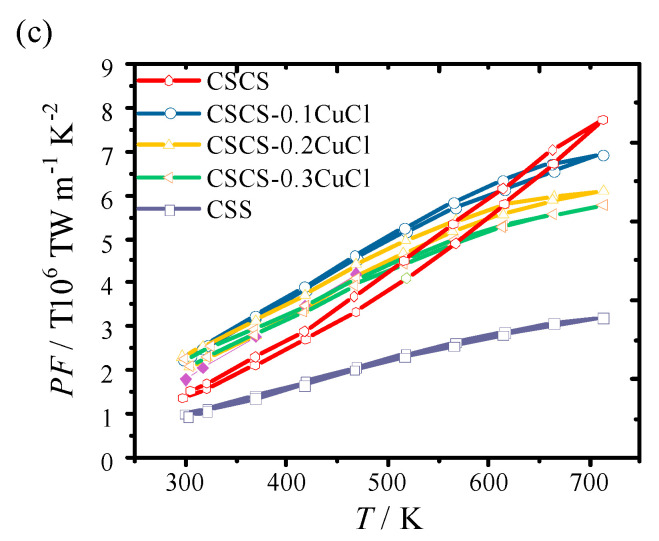
Thermoelectric properties ((**a**) electrical conductivity, σ, (**b**) Seebeck coefficient, *S*, (**c**) power factor, *PF*) as a function of temperature for CSCS-*x*CuCl (*x* = 0, 0.1, 0.2, 0.3) samples.

Figure 4 shows the thermoelectric properties of CuCl-doped samples at one thermal (heating and cooling) cycle. All of the samples showed excellent data repeatability, indicating superior chemical stability at high temperatures of up to 700 K. For the pristine CSS phase, σ reached 150 S cm^−1^ at room temperature, which is much higher than other reported values [14] using different synthesis methods. Compared to the reported work using the melting ingot method [14,15], σ of the pristine phase showed a much lower value of ~5 S cm^−1^, which was highly attributed to the preservation of S in the monoclinic phase. Other methods such as the ball milling method [13] or the method employed in this study (a combination of ball milling and hot-pressing in vacuum) could largely release S during the sintering progress. The influence of sintering conditions on the thermoelectric performance of chalcogenides has also been systematically studied in our previous work [25]. The study revealed that the repeatability of the thermoelectric data would be improved under the maximum testing temperature. Alternatively, due to the evaporation of S or Se during the sintering or testing progress, the sample turned chemically stable, which favors the data repeatability. Furthermore, it can be seen that all the samples showed the metal-like conduction behavior, which is completely different when compared to the ones using the melting method [15]. With the introduction of Co at the Sn site, the three-dimensional states of Co enhanced the density of the state and boosted the room-temperature σ to ~3000 S cm^−1^, which is almost double the reported value of CSCS from Zhao et al. [12]. The enhanced σ are also attributed to the large copper vacancy introduced by the Co dopant. The proposed solid solution formation with Co doping is described in Formula (1) with the analysis of the XRD profile. In addition to the doping effect of Co, the derived conductive precipitates, such as CoS_2_, Cu_1.8_S, and SnS, are taken into account of large σ [14]:

(1) $${\mathrm{Cu}}_{2}{\mathrm{SnS}}_{3}\overset{x\mathrm{Co}}{\to }\;\underset{\begin{array}{c} original\;\\ Cu-site \end{array}}{\underset{︸}{\left({\mathrm{Cu}}_{2-1.8w}{☐}_{1.8w}\right)}}\underset{\begin{array}{c} original\;\\ Sn-site \end{array}}{\underset{︸}{\left({\mathrm{Sn}}_{1-z}{\mathrm{Co}}_{y}\right)}}\underset{\begin{array}{c} original\;\\ S-site \end{array}}{\underset{︸}{\left(S_{3-2y-z-w}{☐}_{2y+z+w}\right)}}\;+\underset{precipitate}{\underset{︸}{y{\mathrm{CoS}}_{2}+z\mathrm{SnS}+w{\mathrm{Cu}}_{1.8}S}}\;+\underset{carrier}{(\underset{︸}{2y+z+w)\;electron+1.8w\;hole}}$$

In contrast, with the introduction of CuCl, the reduction of Cu vacancy density and the elimination of holes are considered as highly possible outcomes. Thus, the Cu vacancy and hole concentration could be tuned simultaneously by modifying the doping amount of CuCl as demonstrated by EDX analysis. Meanwhile, Cl doping to the *S* site also acts as an acceptor to reduce the carrier concentration and dampen the σ. Such an effect could be demonstrated in other copper-based chalcogenides, such as Cl-doped Cu_2_Se [24].

As shown in Figure 4a, the introduction of 10 mol% CuCl dampened σ to almost half of the original value for the CSCS sample. With increasing *x* to 0.3, σ approached saturation at ~600 S cm^−1^ which is quite close to the optimized σ for most modified CSS thermoelectric systems [14]. The *PF* of the pristine CSS showed a linear relationship against the testing temperature with a peak of ~3 μW cm^−1^ K^−2^. The Co modified sample showed the highest *PF* at 713 K, while the CuCl-doped samples demonstrated the enhancement of *PF* at the temperature range from 300 K to 600 K.

**Figure 5:**
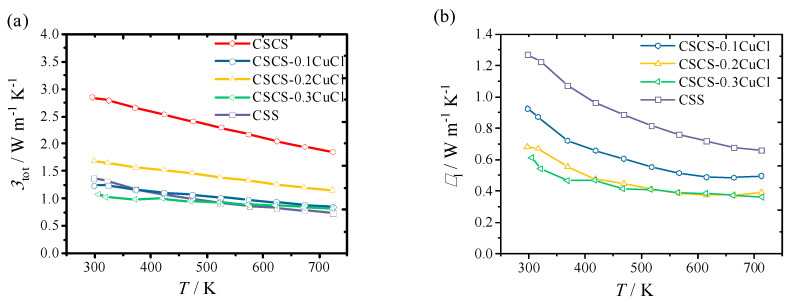
Thermal transport properties: (**a**) total thermal conductivity, $\kappa_{tot}$, and (**b**) lattice thermal conductivity, $\kappa_{l}$, as a function of temperature for CSCS-*x*CuCl (*x* = 0, 0.1, 0.2, 0.3) and pristine CSS samples.

Figure 5 shows the total thermal conductivity,$\;\kappa_{tot}$, and lattice thermal conductivity,$\;\kappa_{l}$, as a function of testing temperature. As shown in Figure 5a, $\kappa_{tot}$ for all the samples decreased with increasing temperature. $\kappa_{tot}$ for the pristine CSS sample ranged from 1.36 W m^−1^ K^−1^ to 0.738 W m^−1^ K^−1^, which is comparable to the reported values prepared by other methods [13,14]. To suppress the phonon transport in the pristine CSS material, the Co element with half of the atomic weight of Sn was employed in this study. The effect of alloying with Sn sites on $\kappa_{lat}$ can be analyzed by the Wiedemann-Franz Law. However, due to the strong electron-electron interaction, it is difficult to extract $\kappa_{lat}$ from $\kappa_{tot}$ for the CSCS sample. This issue can also be encountered by those semiconductors with high carrier concentration and a low Seebeck coefficient. Finally, the sample substituted with *x* = 0.3 displayed the highest $\kappa_{tot},$ ranging from 2.84 W m^−1^ K^−1^ to 1.85 W m^−1^ K^−1^. The values are much higher when compared to the reported work in which Zhao et al. [12] demonstrated that $\kappa_{tot}$ ranged from 2.0 to 1.5 W m^−1^ K^−1^ with the doping of 25 mol% Co. This discrepancy can be explained by the compositional deviation of the bulk sample due to the loss of Cu during the mixing process, introducing a large value of $\kappa_{e}$. Though the influence of Cu site vacancy on the carrier transport properties has not been studied, the analogy between Cu_2_SnS_3_ and Cu_2_SnSe_3_ is reasonable [26]. According to Cheng et al., the existence of the Cu vacancy would enhance the carrier density and shift the Fermi level deeply into the valence band, exhibiting the multiband structure [27]. Furthermore, the heavy doping of Co might tune the energy band, in which the Co-3d states significantly contribute to the density of states. With the existence of CuCl, strong phonon scattering occurred over the testing temperature range. An obvious drop in $\kappa_{tot}$ from 2.84 to 1.23 W m^−1^ K^−1^ was obtained at room temperature.

To have a better understanding of the doping effect on phonon scattering, $\kappa_{l}$ was calculated by subtracting the carrier thermal conductivity from $\kappa_{tot},$ as shown in Figure 5b. According to the Wiedemann-Franz Law, the carrier contribution to thermal conductivity is related as *LσT*:

(2) $$L=\frac{k_{B}{}^{2}}{e^{2}}\frac{\left(1+\lambda \right)\left(3+\lambda \right)F_{\lambda }\left(\eta \right)F_{\lambda +2}\left(\eta \right)-{\left(2+\lambda \right)}^{2}F_{\lambda +1}{\left(\eta \right)}^{2}}{{\left(1+\lambda \right)}^{2}F_{\lambda }{\left(\eta \right)}^{2}}$$

where *L* is the Lorenz number, $k_{B}$ is the Boltzmann constant, and *F_i_* is the Fermi-Dirac integral of *i*th order (*F_i_* = $\int_{0}^{\infty }x^{i}/\left(1+\exp\left[x-\eta \right]\right)dx$)). The carrier mobility is assumed to be limited by acoustic phonon scattering (λ=0), and the reduced chemical potential η is obtained as a function of temperature from the experimental Seebeck coefficients using the equation below [25,28]:

(3) $$S=\frac{k_{B}}{e}\left(\frac{\left(2+\lambda \right)F_{\lambda +1}\left(\eta \right)}{\left(1+\lambda \right)F_{\lambda }\left(\eta \right)}-\eta \right)\;$$

$\kappa_{l}$ for the CSCS sample is excluded in Figure 5b because of the strong electron-electron interaction. The minimum $\kappa_{l}$ of the pristine CSS sample was 0.65 W m^−1^ K^−1^, while that of CSCS-*x*CuCl (x = 0.2, 0.3) was 0.36 W m^−1^ K^−1^. This value is close to the theoretical minimum value of 0.3 W m^−1^ K^−1^. Thus, the ultralow $\kappa_{l}$ was demonstrated by multiple phonon scattering, including alloying scattering and impurity scattering in different scales.

As shown in Table 2, after being doped with CuCl, the speeds of sound are reduced compared to those of the pristine CSCS and CSS samples. The tendency of speeds of sound in Table 2 corresponds well to the trend in Figure 5b. The same trend was found from the variations of Young’s modulus, shear modulus, and the adiabatic bulk modulus, which indicated the interatomic force softening [14]. Among all the modified compositions, the speeds of sound and mechanical moduli for CSCS-0.3CuCl show the minimum values.

**Figure 6:**
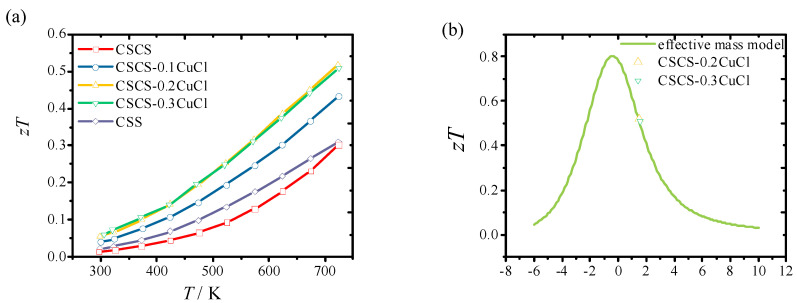
(**a**) Figure of merit, *zT*, as a function of temperature for the pristine CSS and CSCS-*x*CuCl (*x* = 0, 0.1, 0.2, 0.3) samples and (**b**) effective mass model derived *zT* against the reduced chemical potential at 700 K for CSCS-*x*CuCl (*x* = 0, 0.2, 0.3) samples.

Figure 6 shows the calculated figure of merit, *zT*, as a function of temperature, in which *zT* increased with temperature for all samples. Both the sample of CSCS-0.3CuCl and CSCS-0.2CuCl shows optimized performance, which is almost 200% compared to the pristine sample CCS. To be frank, the *zT* was evaluated using the instrument without the cold finger effect. A fair comparison needs to be conducted with a scaling factor of 1.2 times our maximum *zT* value. The maximum *zT* of 0.56 was obtained at 700 K for CSCS-0.3CuCl and CSCS-0.2CuCl samples, which is almost double that of pristine samples and comparable to other reported data [11,13]. By applying the effective mass model, the ex[isting *zT* values are located well at the prediction curve of *zT,* as shown in Figure 6b. The *zT* value could be enhanced if we further reduced the carrier concentration using alternative strategies such as the energy filtering method and the introduction of nano inclusions.

## 4. Conclusions

The CuCl-doped CSCS-*x*CuCl (*x* = 0, 0.1, 0.2, 0.3) thermoelectric material system has been successfully synthesized by using the ball milling and hot press sintering method. The proposed synthesis method produces the main CSS phase structure with multiple impurities. It has been demonstrated that the introduction of CuCl could effectively compensate for copper loss, helping boost the *PF* over a wide temperature range. Meanwhile, the reduced lattice thermal conductivity (~0.38 W m^−1^ K^−1^ at 700 K) demonstrated the dampened phonon propagation, which is beneficial for improving the zT value. Finally, the CuCl dopant provides an effective way to enhance the thermoelectric performance of the CSCS system. When the doping amount reached 20 mol%, the maximum *zT* could reach 0.53 at 700 K, which is almost double that of the pristine samples. This zT value is measured using an instrument without the cold finger effect, which is more competitive among reported values. We believe that CuCl is an effective dopant for other copper-based chalcogenides with Cl doping.

## Figures and Tables

**Table 1:** The lattice parameters variation against the excess amount of Cu, *x*, for the CSCS-*x*CuCl (*x* = 0, 0.1, 0.2, 0.3) and CSS samples.

	a/Å	b/Å	c/Å	angle/°	$V/{Å}^{3}$
CSCS	5.3960	5.3960	10.8266	90	315.2361
CSCS-0.1CuCl	5.4233	5.4233	10.8497	90	319.1133
CSCS-0.2CuCl	5.4212	5.4212	10.8481	90	318.8192
CSCS-0.3CuCl	5.4211	5.4211	10.8481	90	318.8192
CSS	5.4387	5.4387	10.8475	90	320.8631

**Table 2:** Room-temperature speeds of sound and calculated mechanical properties for CSCS-*x*CuCl (*x* = 0, 0.1, 0.2, 0.3) and CSS samples.

Composition	ρ	$\upsilon_{L}\;ms$	$\upsilon_{T}\;ms$	$\upsilon_{m}\;ms$	*G* GPa	*E* GPa	*Bs* GPa
CSS	4.71	4714	2357	2644	26.2	69.7	69.7
CSCS	4.78	4866	2336	2627	26.1	70.4	78.4
CSCS-0.1CuCl	4.76	4703	2267	2548	24.5	65.9	72.6
CSCS-0.2CuCl	4.54	4586	2259	2536	23.2	62	64.6
CSCS-0.3CuCl	4.61	4387	2167	2432	21.6	57.9	59.8

Remarks: $\upsilon_{L}$ is the longitudinal speed of sound, $\upsilon_{T}$ is the transverse speed of sound, $\upsilon_{m}$ is the calculated mean speed of sound ($\upsilon_{m}$ = 3^1/3^ ${\left(\upsilon_{L}^{-3}+2\upsilon_{T}^{-3}\right)}^{-1/3})$ , *G* is the shear modulus, *E* is Young’s modulus, and *Bs* is the adiabatic bulk modulus.

## Data Availability

Data is contained within the article.
